# Patient Eczema Education Pictorial Study (PEEPS): A Pilot Investigation

**DOI:** 10.1177/12034754251320645

**Published:** 2025-03-12

**Authors:** Bethany F. Wilken, Sonja Molin, Thomas Herzinger, Robert Bobotsis, Anne K. Ellis, Yuka Asai

**Affiliations:** 1 Division of Dermatology, Department of Medicine, Queen’s University, Kingston, ON, Canada; 2 Division of Dermatology, Temerty Faculty of Medicine, University of Toronto, Toronto, ON, Canada; 3 Division of Allergy and Immunology, Department of Medicine, Queen’s University, Kingston, ON, Canada

**Keywords:** atopic dermatitis, patient education, quality of life, self-management, disease severity

## Abstract

**Background:**

For optimal control of atopic dermatitis (AD), patient education is essential to complement traditional therapy. Patient education has proven to benefit AD outcomes, but previous methods of delivery are costly and time-consuming.

**Objective:**

To assess the effectiveness of a one-page pictorial education tool at improving AD quality of life (QoL) and disease severity.

**Methods:**

Patients with AD and caregivers (if patient <18 years) received education with a pictorial education tool. QoL and disease severity were measured at baseline and in follow-up 2 to 6 months after education.

**Results:**

Forty-seven patients and caregivers from speciality clinics in dermatology and allergy received education. At follow-up, there were significant decreases in QoL scores and median disease severity scores.

**Conclusions:**

A pictorial education tool for AD is associated with significant benefits for patients and caregivers after 2 to 6 months. This tool may be valuable for health care providers who are in need of an effective and efficient method of AD education; however, further studies are needed to address identified knowledge gaps and expand to other sites and non-specialist clinics.

## Introduction

Atopic dermatitis (AD), also known as eczema, is a chronic inflammatory skin disease that has a pronounced impact on quality of life (QoL) for patients and their families.^1-3^ AD is often a life-long, relapsing-remitting disease; thus, patients must have knowledge of the pathogenesis, triggers, preventative measures and the need for prompt treatment to enable successful self-management. A lack of understanding of the disease process and management strategy can contribute to poor clinical outcomes.^4^ In chronic diseases, only 2% of care occurs in the clinic; therefore, the goal of patient education is to teach patients the knowledge and skills required to manage the remaining 98% of care.^5^ AD guidelines recommend education as part of therapy,^6-10^ yet patient education practices are diverse in delivery and content.

Various studies have shown significant positive results for AD patient education,^11,12^ with most improvements seen in knowledge, disease severity and QoL.^4,13-19^ Patient education supports an increased understanding in treatment regimen and a subsequent decrease in confusion and frustrations towards management.^4,13,17,20-22^ Patient empowerment is a key indicator of a patient’s capacity for responsible self-handling of chronic diseases.^23^ Education facilitates empowerment in patients with AD through measures of self-efficacy and confidence in self-care.^16-18,24^

Methods of delivery for patient education in AD differ and may require extensive resources, which limits their widespread implementation. Group programming with an interdisciplinary team or individual consultations with a health care professional are valuable,^13,14,16,17,21,25,26^ but time-consuming and expensive to organize and administer. Missing work or school to attend educational sessions in addition to medical appointments for patients/caregivers and the time and cost for professionals to be trained and to administer the education can be unfavourable, especially as there is a lack of financial assistance from large health authorities (FDA, WHO) who encourage the large-scale development of eczema educational centres.^27^ The COVID-19 pandemic has further exhausted clinic resources and drained health care personnel,^28^ highlighting the pressing need for educational interventions to be both cost- and time-effective.

Paper handouts or online educational resources that are readily accessible and distributable could decrease the cost:benefit ratio of education. Some educational handouts have shown benefit,^20,22,24^ but others may be unsuitable and are often written at a literacy level higher than is recommended for the lay public.^29^ We therefore developed and evaluated a one-page, printable handout of a pictorial education tool in order to decrease health literacy burden and stimulate reader engagement. The tool was deliberately designed to be convenient for use in clinics with limited resources, and not require technological equipment or additional personnel. The outcome parameters followed previous AD education studies by focusing on clinical severity and QoL scores.

## Methods

### Participants and Study Design

This pre-post pilot study was approved by the Health Sciences Research Ethics Board of Queen’s University, Kingston, ON, Canada. Forty-seven patients and/or caregivers with mild-to-severe AD were recruited from a dermatology and multidisciplinary allergy and dermatology clinic. Most participants were recruited in the spring/summer, and follow-up commonly took place in the fall and winter (Supplemental Table 2). Patients of all ages were recruited (and caregivers if <18 years). The inclusion criteria required participants to have a clinical diagnosis of AD. Patients were excluded if they had a clinical diagnosis of dermatitis not classified as atopic (eg, contact dermatitis). Previous AD treatments, disease severity and time with disease did not influence eligibility. All participants were required to comprehend and complete the informed consent. Participants gave written informed consent, and children aged 7 to 14 years gave written assent. The participants and educators could not be blinded as they were aware that they were receiving/using the education tool. Participants were assessed at baseline, and 2 to 6 months after education.

### Intervention

The education tool was written at a sixth-grade reading level and had simple, cartoon-style graphics of diverse skin types (Supplemental Figure 1). It is deliberately non-prescriptive to ensure uptake and adaptability for each practitioner and patient care plan. The tool is based on ‘*eczema as a wildfire*’ analogy that has long been used by many dermatologists. While practitioners were not told exactly how to use the tool, the components of the tool included the following:

*At risk of fire*: Introduces the known association of AD with dry skin, and how the use of emollients and bathing are key to the *prevention* of AD flares. Dry dead brush is a prime setting for a wildfire, just as keeping the skin moisturized is key for preventing AD flares.
*Spark a fire*: Discusses common *triggers* (ie, irritants, stress, illness) that can activate an AD flare, similar to how sparks ignite a wildfire.
*At first sight, put out the fire*: Encourages early intervention to treat flares before they worsen, using topical treatments like a *fire extinguisher*.
*. . .Completely*: Stresses the need to continue treatment until the flare is fully resolved to prevent recurrence.
*Prevent the fire recurrence*: Introduces maintenance therapy (eg, non-steroidal creams) as a fire retardant to prevent future flares.
*Send for reinforcements when needed*: Guides the use of *systemic therapies* for severe AD that doesn’t respond to topical treatments.

After obtaining informed consent and completing the study questionnaires, the intervention of the pictorial education tool was performed the same day during the patient’s scheduled appointment. The participant’s dermatologist (n = 2) went through the handout with the patient/caregiver, focusing on relating care recommendations to each stage of the ‘wildfire’. If a resident (n = 1) or research team member (n = 1) used the tool, care recommendations strictly followed the ones given by the patient’s doctor, and treatment plans were not altered. Each patient and their family were able to take home a copy of the pictorial education handout for future reference.

### Outcome Measures

It is suggested that the assessment of patient education for AD should include a biomedical outcome, QoL scores and specific psychological scores.^27^ On the basis of this suggestion, we chose to measure QoL and clinical severity. At the time this study was conducted, there were no validated tools to measure AD knowledge.

I. Quality of life was assessed with the Dermatology Life Quality Index (DLQI),^30^ the Children’s Dermatology Life Quality Index for 5 to 16 years^31^ and the Infants’ Dermatitis Quality of Life Index for 4 years and under.^32^
II. Disease severity was measured using the Eczema Area and Severity Index (EASI).^33^ Disease severity was stratified into clear/almost clear (EASI score 0-1), mild (EASI score 1.1-7.0), moderate (EASI score 7.1-21.0), severe (EASI score 21.1-50.0) and very severe (EASI score 51.1-72.0).

### Statistical Analysis

All statistical evaluations were carried out using RStudio (version 1.3.1093; Integrated Development Environment for R, PBC, Boston, MA, USA). Normality of the data was tested using the Shapiro-Wilk test. Normally and non-normally distributed paired data were analyzed using the paired *T*-test and the Wilcoxon signed-rank test, respectively. For all tests, statistical significance was set at .05.

## Results

**Figure 1.**
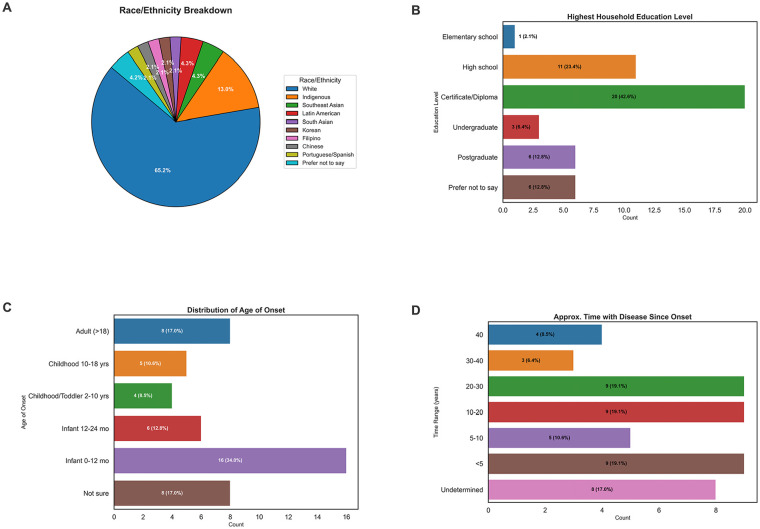
Demographic and clinical characteristics of study participants. (A) Race/ethnicity breakdown of study participants (n = 47). (B) Highest household education level of study participants (n = 47). (C) Distribution of age of onset of AD in study participants (n = 47). (D) Approximate time of disease since onset in study participants (n = 47). AD, atopic dermatitis.

Thirty-one adults with AD and 16 children with AD and their caregivers (mean age 31 years (range 13 months-89 years); female 51.1%, male 46.8%, non-binary 2.1%) received education with the pictorial education tool from 4 different providers (dermatologist n = 2, dermatology resident n = 1, research assistant n = 1). The diversity of our study participants (Figure 1; Table 1) exceeded the reported demographic diversity of the city where the study took place (Statistics Canada, 2021) and also had an overall lower level of educational attainment (Figure 1) compared with Canadian estimates (Statistics Canada, 2021). The majority of participants had mild disease (46.5%), followed by moderate (32.6%), clear (9.3%) and severe (9.3%). The average duration of disease of participants since diagnosis was 19 years (<1 to >40 years).

**Table 1.** Demographic and Clinical Characteristics of Study Participants.

Variable	Study participants
Mean age (years)	31 (6 months-91 years)
Adult patients	n = 31
Mean age	42.5 (18-91)
Children	n = 16
Mean age	9 years (6 months-15 years)
Gender	
Male	22 (46.8%)
Female	24 (51.1%)
Non-binary	1 (2.1%)
EASI score (disease severity)	
0-1 (clear/almost clear)	4 (8.5%)
1.1-7 (mild)	20 (42.5%)
7.1-21.0 (moderate)	14 (29.8%)
21.1-50 (severe)	4 (8.5%)
50.1-72.0 (very severe)	1 (2.1%)
Not recorded	4
DLQI (effect on patient’s life)	
0-1 (no effect)	5 (10.9%)
2-5 (small)	6 (13.0%)
6-10 (moderate)	15 (32.6%)
11-20 (very large)	15 (32.6%)
21-30 (extremely large)	5 (10.9%)
Not recorded	1

Abbreviations: DLQI, Dermatology Life Quality Index; EASI, Eczema Area and Severity Index.

Four participants did not have an EASI score recorded at baseline, and one did not complete the DLQI questionnaire; thus, they were omitted from further clinical score analysis. There were 12 participants lost to follow-up for the following reasons: missed follow-up appointment (n = 8), discharged from speciality clinic (n = 2) and other (n = 2) (see population characteristics in Supplemental Table 3).

### QoL and Disease Severity

At 2 to 6 months after education, QoL significantly improved from baseline (Table 2). The median QoL score fell from 10 at baseline to 6 after the implementation of the pictorial handout. Post-intervention, the median of disease severity scores also significantly decreased from 8 (moderate disease) at baseline to 5 (mild disease) 2 to 6 months later.

**Table 2.** QoL and Eczema Severity Scores at Baseline and Follow-Up (2-6 months).

Variables	Scores		*P*-value	
	Mean ± SD	Median (IQR)	Normality	Signed-rank test
QoL (n = 36)				
Baseline	11.0 ± 7.61	10 (9)		
2-6 months	7.58 ± 6.69	6 (10)	.01296*	.00631*
EASI (n = 33)				
Baseline	13.0 ± 13.1	8 (13.1)		
2-6 months	7.97 ± 10.1	5 (11.5)	.00683*	.03679*

Abbreviations: EASI, Eczema Area and Severity Index; QoL, quality of life.

* *P* < .05.

## Discussion

Given the chronicity and treatment complexity of AD, education plays a vital role in its management. Patients and caregivers in our study demonstrated statistically significant improvements in QoL scores and disease severity.

These findings support previous studies in AD that have shown that patient education contributes to improved QoL and clinical outcomes.^13,14,19^ Our pictorial education tool resulted in a significant decrease in DLQI and EASI scores. Notably, the median decrease of 4 in DLQI scores represents a meaningful clinical change.^34^ This is in contrast to prior studies that have evaluated AD educational handouts and have used DLQI and EASI scores as outcome measures and found no statistically significant differences in clinical scores.^20,24^ The use of analogies to describe health issues and visual displays of information can help to improve patient understanding,^35,36^ and the use of illustrations and describing AD as a ‘wildfire’ may help to explain the enhanced efficacy of our tool.

There is debate surrounding whether single interventions are sufficient to improve management of AD and to change patients’ behaviour.^16,25^ Reflected in improved disease severity and QoL at 2 to 6 months, we believe that patients may have been able to transfer the knowledge and attitudes into daily life after a single educational session. Although patients were not specifically asked whether they reviewed the tool at home, we consider that because patients and families were able to take the handout home with them, it may have improved knowledge retention and helped to promote the recall of health-related information. Armstrong et al found that a reviewable video intervention promoted immediate and delayed retention of AD knowledge while significantly decreasing AD severity.^4^ An English psychologist who studied factors affecting patient recall asserted that notable information should be organized into specific categories.^37^ While this study is small and in a population of patients receiving specialist care, it is possible that by relating each AD management factor to the stage of a wildfire, we may have been able to overcome the challenge of poor patient recall in dermatology education.^38^

Assessment of patient education for AD should be multifactorial, including specific knowledge scores.^27^ Previous studies on AD patient education have relied on non-validated tools to assess AD patient knowledge,^13,20-22^ which limit their reliability. This paper shows clinical improvement as a result of educational intervention; to determine what specific knowledge or self-efficacy components lead to this objective clinical improvement is an important area of future study. Such questionnaires can help clinicians identify specific areas where patients may need more education, enabling a more tailored, patient-centred approach to care. Since this study was performed, there has been some development in this area with the Infant Skincare, Knowledge, Attitude, and Practice (ADISKAP) scale,^39^ but given the increasing prevalence of adult-onset AD,^40^ more tools need to be developed that are inclusive of all AD demographics.

While this is a small single-centre study, one strength of this project is the inclusion of participants of all ages, disease severity and time with disease may be representative of patients in real clinical practice. Supporting generalizability, the distribution of patients according to disease severity in this study is similar to proportions of AD severity observed in epidemiological studies.^41,42^ The discord between EASI and DLQI scores, with 76.1% of participants reporting AD had a moderate-very large impact on their QoL versus only 40.4% of participants having moderate-severe disease, also correlates to the limited concordance between QoL and disease severity scores observed in other AD studies.^43,44^ It should be noted that clinical severity was not a general estimate but assessed on the condition of the participant’s skin on that specific day. As a result, some participants may have responded well to treatment but still have severe disease. We included in our study patients diagnosed as having well-controlled AD at the time of visit (‘clear’) but who are known to have severe AD.

It is well known that AD flares are associated with seasonal changes; specifically, many patients get worse in the winter.^45-47^ Flares can be caused by low temperatures in the winter drying out the skin and/or flares can be induced by viral illnesses, which have an increased prevalence during the winter months.^48^ It is reassuring that we were able to detect improved clinic status after education despite following up with participants at a time of year during which their disease may commonly worsen. Nevertheless, some patients experience more flares in the spring and summer.^49^ Future studies could better account for seasonal variations in disease severity by performing recruitment and follow-up at more than one time point.

When interpreting the results of our study, we must consider them within the context of the study design. The efficacy of the tool should be further investigated through a randomized controlled trial, with assessment by a physician blinded to randomization. This will help to substantiate that the positive association between education and improvements in disease severity/QoL was not due to external influences such as treatment changes. This study occurred in a tertiary care setting, but many AD sufferers seek initial treatment from their primary care provider. In fact, around two-thirds of patients have mild disease that can be adequately managed at the primary care level.^50^ A study evaluating its usability and applicability to non-specialists is warranted.

## Conclusion

Patient education is becoming increasingly recognized as an essential component to AD management. Although education delivered through traditional workshops, ‘eczema schools’ and specialized consultations has improved patient outcomes, these methods are costly, time-consuming and require significant organization to be implemented. This study demonstrates a pictorial, one-page, printable handout as a potential efficient and effective educational tool. Participants reported significant improvements in all study endpoints, including QoL and disease severity. Adding to this pilot study, further work is needed to assess the tool with a control group, in primary care, in other academic centres and with follow-ups at multiple time points.

After further refinement, we anticipate the advancement of this tool for broader dissemination within the dermatology community and primary care settings in the near future. This process aims to empower patients while positively influencing clinical outcomes in AD.

## Supplemental Material

sj-docx-1-cms-10.1177_12034754251320645 – Supplemental material for Patient Eczema Education Pictorial Study (PEEPS): A Pilot Investigation Supplemental material, sj-docx-1-cms-10.1177_12034754251320645 for Patient Eczema Education Pictorial Study (PEEPS): A Pilot Investigation by Bethany F. Wilken, Sonja Molin, Thomas Herzinger, Robert Bobotsis, Anne K. Ellis and Yuka Asai in Journal of Cutaneous Medicine and Surgery
