# Supplementary material for: Patient Eczema Education Pictorial Study (PEEPS): A Pilot Investigation
Source: J Cutan Med Surg. 2025 Mar 12;29(4):374–80. doi: 10.1177/12034754251320645 (PMC12304491; doi:10.1177/12034754251320645)
Supplement: sj-docx-1-cms-10.1177_12034754251320645 – Supplemental material for Patient Eczema Education Pictorial Study (PEEPS): A Pilot Investigation [file sj-docx-1-cms-10.1177_12034754251320645.docx]

Supplementary Material

Table S1. The number of participants recruited, and follow-ups performed in each month.

| ***Month*** | ***# of participants recruited*** | ***# of follow-ups*** |
| --- | --- | --- |
| May | 10 | n/a |
| June | 9 | n/a |
| July | 9 | 1 |
| August | 3 | 1 |
| September | 6 | 3 |
| October | 8 | 9 |
| November | 2 | 4 |
| December | n/a | 5 |
| January | n/a | 4 |
| February | n/a | 5 |
| March | n/a | 3 |
| April | n/a | 1 |

Table S2. Baseline characteristics comparing included participants vs. those lost to follow-up.

|  | **Included (n=47)** | **Lost to follow-up (n=12)** |
| --- | --- | --- |
| *Adults* | n=31 | n=8 |
| *Children* | n=17 | n=3 |
| **Mean age** | 31yrs (6mo-91yrs) | 32yrs (11yrs-69yrs) |
| **Gender (M/F/NB)** | 22/24/1 | 5/6/1 |
| **QoL**  (% of cohort) | no effect- 10.9%  small effect- 13%  moderate effect- 32.6%  very large effect- 32.6%  extremely large effect- 10.9% | no effect - 20%  small effect- 10%  moderate effect- 20%  very large effect- 40%  extremely large effect- 10% |
| **Disease severity**  (% of cohort) | Clear/almost clear- 8.5%  mild- 42.5%  moderate-29.8%  severe-8.5%  very severe-2.1% | Clear/almost clear- 30%  mild- 30%  moderate- 20%  severe- 20%  very severe- 0% |
| **Highest household education level**  (% of cohort) | Elementary school-2.1%  High school- 23.4%  Certificate/Diploma- 42.5%  Undergraduate- 6.4%  Postgraduate- 12.8%  Prefer not to say- 12.8% | Elementary school- 16.7%  High school- 25%  Certificate/Diploma- 33.3%  Undergraduate- 8.3%  Postgraduate- 8.3%  Prefer not to say- n/a |
| **Race/Ethnicity**  (% of cohort) | White- 65.2%  Indigenous- 13%  Southeast Asian- 13%  Latin American- 4.3%  South Asian- 2.1%  Korean- 2.1%  Filipino- 2.1%  Chinese- 2.1%  Portuguese/Spanish- 2.1%  Prefer not to say- 4.2% | White- 66.7%  Indigenous- 25%  Southeast Asian- 0  Latin American- 8.3%  South Asian- 0  Korean- 0  Filipino- 0  Chinese- 0  Portuguese/Spanish- 0  Prefer not to say- n/a |


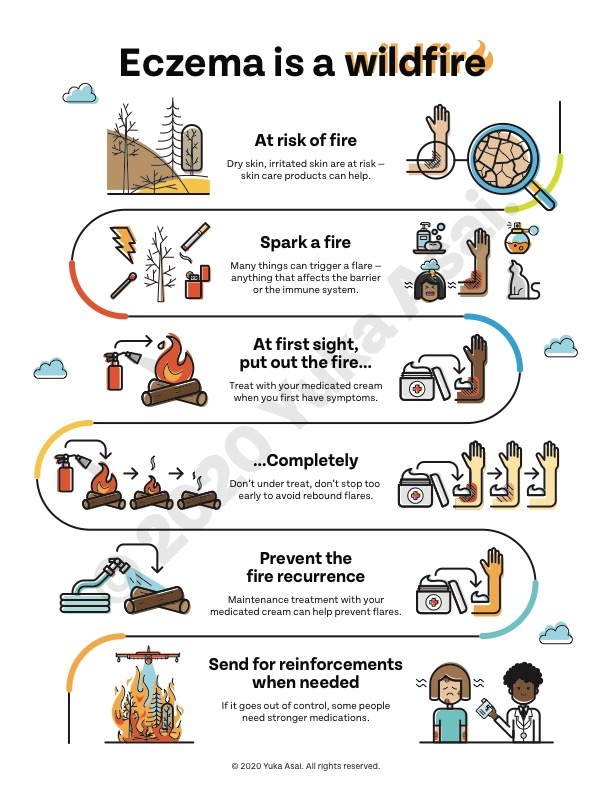
**Figure S1**. English version of the pictorial education tool.


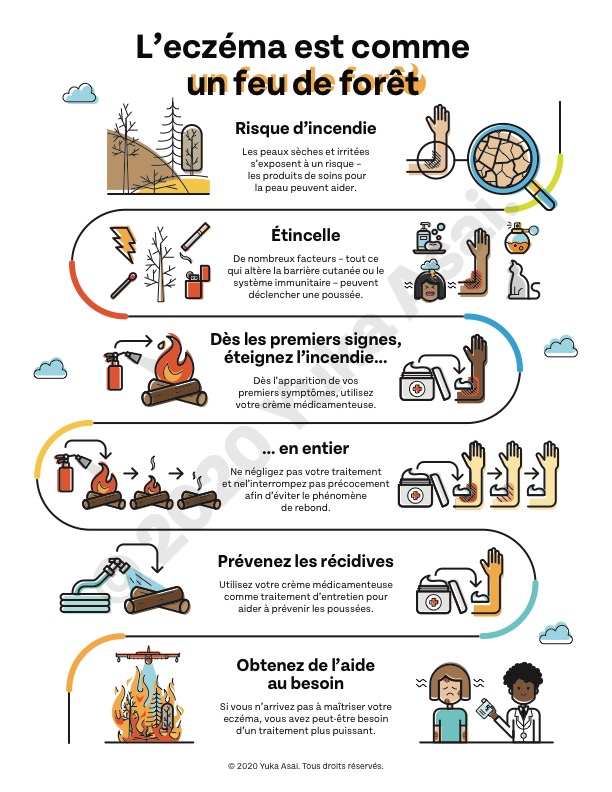


**Figure S2.** French version of the pictorial education tool.
